# Cardio-Oncology: Learning From the Old, Applying to the New

**DOI:** 10.3389/fcvm.2020.601893

**Published:** 2020-11-25

**Authors:** Jun-ichi Abe, Syed Wamique Yusuf, Anita Deswal, Joerg Herrmann

**Affiliations:** ^1^Department of Cardiology, University of Texas MD Anderson Cancer Center, Houston, TX, United States; ^2^Department of Cardiovascular Medicine, Mayo Clinic, Rochester, MN, United States

**Keywords:** evidence-based medicine, type 1 and type 2 cardiotoxicity, anthracycline, trastuzumab, onco-cardiology, cardio-oncology

## Abstract

The recent surge in cancer drug approval has provided us in cardio-oncology with a new and unique era, which modern medicine has not experienced before: the diminishing availability of “conventional” evidence-based medicine. The drastic and quick changes in oncology has made it difficult, and at times even impossible, to establish a meaningful evidence-based cardio-oncology practice by simply following the oncologists' practice. For the modern cardio-oncologist, it seems that a more proactive approach and methodology is needed. We believe that only through such an approach (learn from the old, and apply to the new) the cardio-oncologist will obtain meaningful evidence to perform their every-day practice in this new era.

## Introduction

Over the last few years, the field of cardio-oncology has seen a number of new developments, challenges, and changes in paradigms. First, with advancements in cancer therapy, long-term survival has reached an all-time high. In 2016, in the U.S. alone, the cancer survivor population increased to over 15 million with a projection to over 26 million by 2040. Of further interest, nearly three quarters of these patients are 65 years and older with a higher burden of comorbidities, most frequently cardiovascular in nature (1, 2). Cancer and cardiovascular disease (CVD) notably share risk factors, for example, aging, smoking, and obesity. Also, not only has cancer and its treatment been linked to CVD, but certain CVD conditions, such as heart failure and myocardial infarction, have been linked to an increased risk of cancer as well (3–7). Thus, there is an undeniable and unavoidable intersection of cancer and CVD. While basic and translational research has delineated some of the pathophysiological details in this area, much is still unknown (research lag and gap) and a number of best practice aspects are not defined (practice and education lag and gap).

## Where do We Come from? Changing Paradigms

Historically, much in the field of cardiotoxicity has focused on anthracyclines and trastuzumab. This did provide important insight into mechanisms and principles of how cancer treatments can negatively affect cardiac function. Ewer and Lippman introduced the concept of type 1, irreversible, and type 2, reversible, cardiotoxicity (8). The post-erchild for the first type are anthracyclines such as doxorubicin, which cause dose-dependent injury to the myocardium by many different mechanisms, one of the most important being induction of oxidative stress with related modifications of lipid, protein, and genetic molecules (9, 10). Anthracyclines show high affinity for metal ions, and reduction of the quinone moiety in complex with Fe3^+^ may accelerate reactive oxygen species (ROS) formation (Fenton reaction) (11). Another mechanism is the interaction of anthracyclines with topoisomerase 2β (TOP2β). Anthracyclines bind to both, DNA and TOP2, to form the DNA cleavage complex, which blocks DNA replication, inducing DNA double strand breaks and cell death. TOP2α is highly expressed in proliferating cells such as cancer cells and accounts for the therapeutic action. On the contrary, TOP2β is expressed in the myocardium and leads to the cardiotoxic action (12).

In distinction to the above, initial experience with trastuzumab outlined the occurrence of a different type of cardiac dysfunction (type 2) that was dose-independent and mostly reversible (13–17). Trastuzumab targets one of the outer domains of the HER-2/Erb2receptor, which is overexpressed in ~20–25% of breast cancers and confers a poor prognosis, thus granting pivotal significance to directed therapies (13, 17). Cardiotoxicity, however, remains one of the most important side effects that can be therapy-limiting and therapy-terminating. Intriguingly, this side effect was not anticipated as the role of HER-2/Erb2 signaling in the myocardium was not known at the time. In fact, it was the introduction of trastuzumab that has led to the recognition of HER-2/Erb2 signaling as a myocardial stress response pathway, which is activated and crucial under high afterload conditions and anthracycline exposure (18).

Recently, arguments against the type 1 vs. type 2 concept have arisen, in view of the fact that doxorubicin-induced cardiotoxicity is not always irreversible (9, 10), and on the other hand, trastuzumab-induced cardiotoxicity is not always reversible (19). Indeed, it might be more a matter of the type, timing, duration, and combination of drugs given within the broader context of the patient's genetic and comorbidity profile that determines the trajectory of cardiotoxicity. Indeed, overlap is not inconceivable, and in fact, the sequential and concurrent stress of anthracyclines and trastuzumab has been well-documented. In this context it should be noted that the effects of anthracyclines and trastuzumab on vascular components may be underestimated (20). Doxorubicin can impact endothelial cells, vascular smooth muscle cells, and monocyte/macrophages (20–22). Likewise, trastuzumab treatment can induce a M1-like macrophage phenotype (23) and endothelial dysfunction (24). These vascular aspects may contribute to and complicate the interpretation of cardiotoxicity due to anthracyclines or trastuzumab (25, 26).

**Figure 1:**
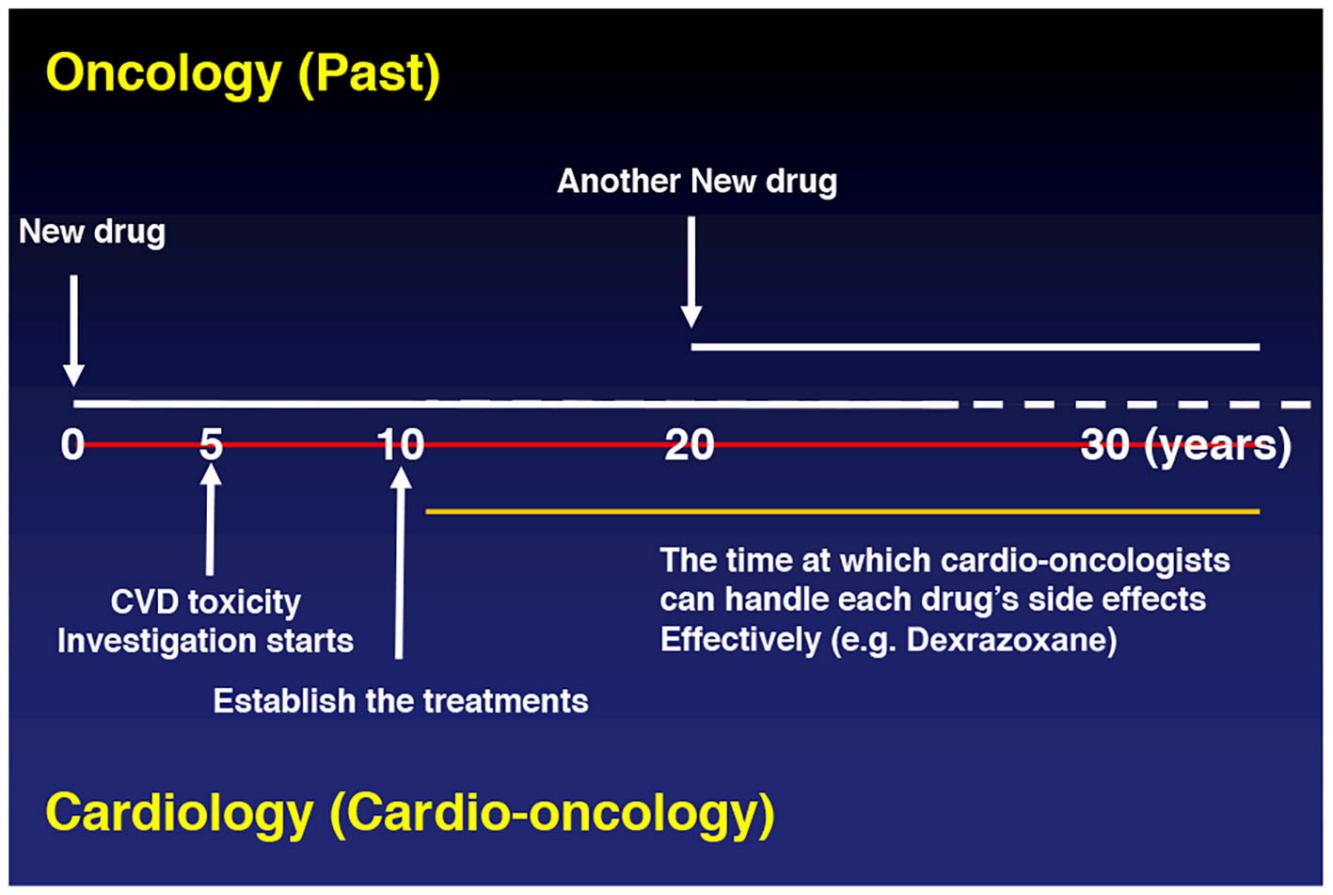
Past.

For the reasons outlined, any subtyping of cardiotoxicity is inherently difficult. While one may argue about some overlap between the type 1 and type 2 cardio-toxicities (27), the concept did, however, help move the field forward, and developing and testing principles and theories will remain key in the emerging discipline of Cardio-Oncology [Figure 1; (28)].

A number of other intertwined molecular processes induced by various cancer treatments, including mitochondrial-related defects [e.g., iron accumulation (11, 29), impaired biogenesis and clearance (30)], degradation of myofilaments (31), and alteration of survival pathways (32), have been suggested, and are discussed extensively in other reviews (25, 33, 34). These multifactorial process and mechanisms, leading to cardiovascular disease in cancer survivors, complicate further the understanding of the pathophysiology of various cancer treatment-induced cardiovascular toxicities, and make it difficult to translate those basic findings into an optimized clinical practice.

## Who are We? Changing Times

Cancer drugs account for 27% of all new drugs in the United States since 2010, with the FDA having approved a total of 126 cancer drugs to treat solid and hematologic tumors from 1980 through 2018 (https://ascopost.com/news/september-2019/cancer-drugs-account-for-over-a-quarter-of-all-new-drug-approvals-in-the-us/). In view of this surge of new therapies, the recent practice of oncology has been changing very frequently and dramatically. One of the most impressive revolutionary milestones in this regard is the development of immune checkpoint inhibitors (ICIs). Currently, hundreds of phase I, II, and phase III/IV clinical trials of anti-PD-1, PD-L1, and CTLA-4 are being carried out across the globe (35). Another ground-breaking development in the area of immuno-oncology has been that of chimeric antigen receptor (CAR) T-cell [and biphasic T cell engager (BITE)] therapy. Furthermore, many chemical inhibitors targeting key DNA damage response proteins, including DNA-PKs (DNA-dependent protein kinase, catalytic subunit), ATM/ATR (ataxia-telangiectasia mutated and Rad3-related), the MRN (MRE11-RAD50-NBS1) complex, and the PARP (poly(ADP-ribose) polymerase) family are emerging as promising cancer therapies (36). On a flip side, the utilization of classical chemotherapeutics or their doses is now diminishing, e.g., anthracycline use in breast cancer.

**Figure 2:**
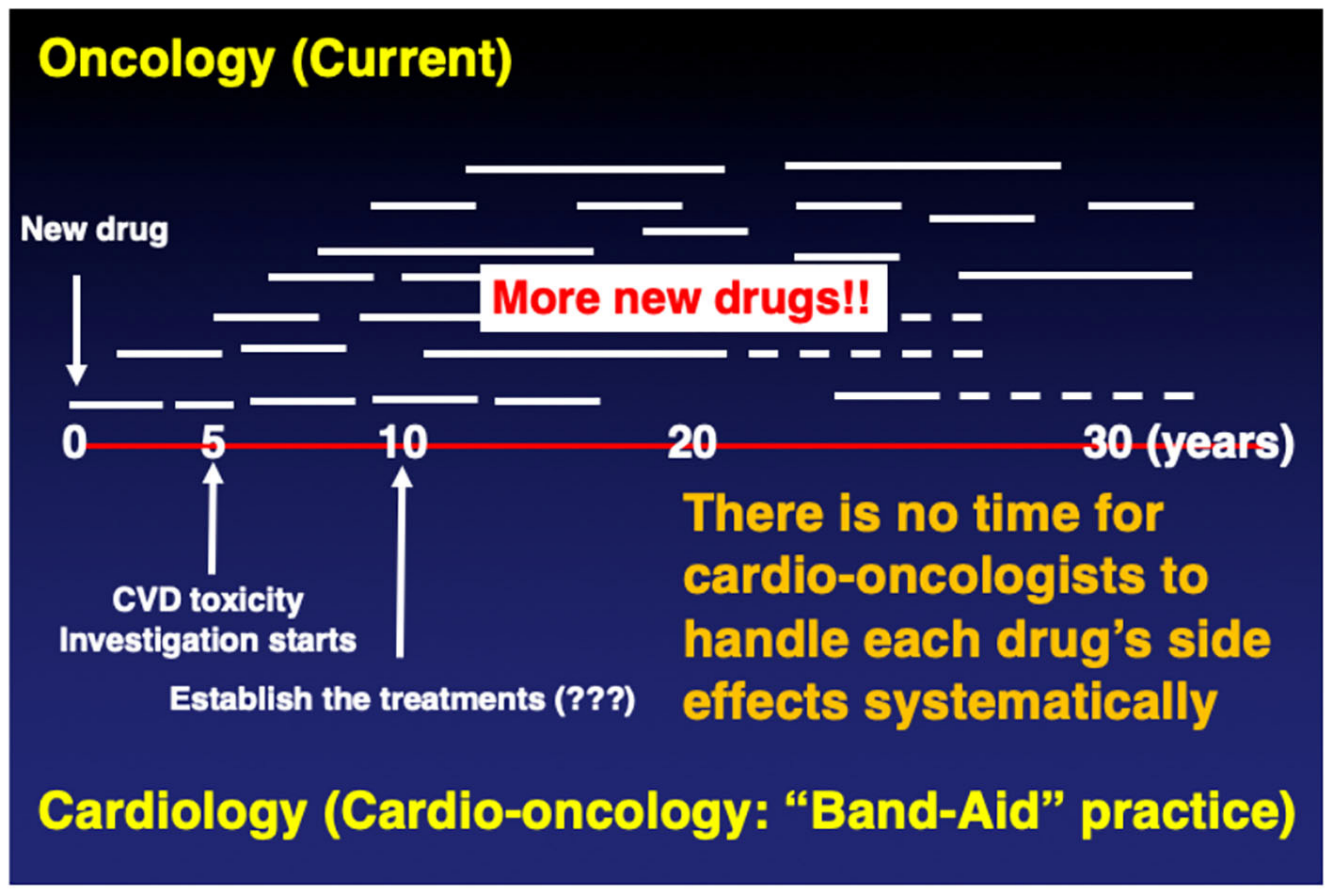
Current.

While these developments illustrate advancements with the goal of improved outcomes, they also point to new challenges. In particular, the scope of oncology is so broad and specialized at the same time with newly emerging drugs and changing practice patterns that no time is given to cardio-oncologists to adapt and generate the evidence necessary for the best management or even prevention of cardiovascular toxicities. For example, if a particular cancer drug is no longer used by the time a cardio-oncologist can define its cardiovascular toxicity profile, management, and prevention approach, those discoveries and insights will be less meaningful. The oncologist will not wait for us (Figure 2).

This being said, some cardio-oncology practice aspects will remain strong, grounded in the general principles of cardiovascular medicine. For example, the management of pre-existing CVD will always have an important role in preventing cardiovascular events. Along those lines, cardio-oncologists are relying on the knowledge of general cardiology and internal medicine. However, general CV principles and treatment of comorbidities will not be enough, and it is necessary to add specifics that are nuanced for the particular agents. Due to the rapid changes and the shortage of time, the current cardio-onclogists are forced to practice without knowing the specificities and without adequate evidence, using a form of “Band-Aid” practice (Figure 2).

## Where are We Going? Changing Minds

In view of the outlined developments there is a need to prepare cardio-oncology specialists for the current and future developments. A key need is structured training in cardio-oncology with core knowledge and core competency requirements. The cardiology fellowship should have a dedicated curriculum for Cardio-Oncology so that even the general cardiologist can have a better understanding of cancer patients afflicted with cardiovascular diseases. Cardiologists sub-specializing in this field should spend at least 6–12 months of clinical training in this field, followed by a subspecialty examination. An initial outline of a cardio-oncology training program has been drafted by the American College of Cardiology, and a board-style certification examinations has been launched by the International Cardio-Oncology Society (ICOS). Other societies and regulatory institutions should follow with the goal of providing nationally and even universally accepted credentials (e.g., the American College of Cardiology and the American Board of Internal Medicine). Aligned with these efforts is the need for standardization of the definition of cardiovascular toxicities in a joint, cross-disciplinary manner. Such initiatives will help clinical practice as well as research, allowing for the direct comparison of outcomes. Furthermore, there should be standardization not only in terms of outcomes but also in terms of availability of cardio-oncology services. Quality and outcomes are another aspect that will be key to further substantiate the importance of the field.

**Figure 3:**
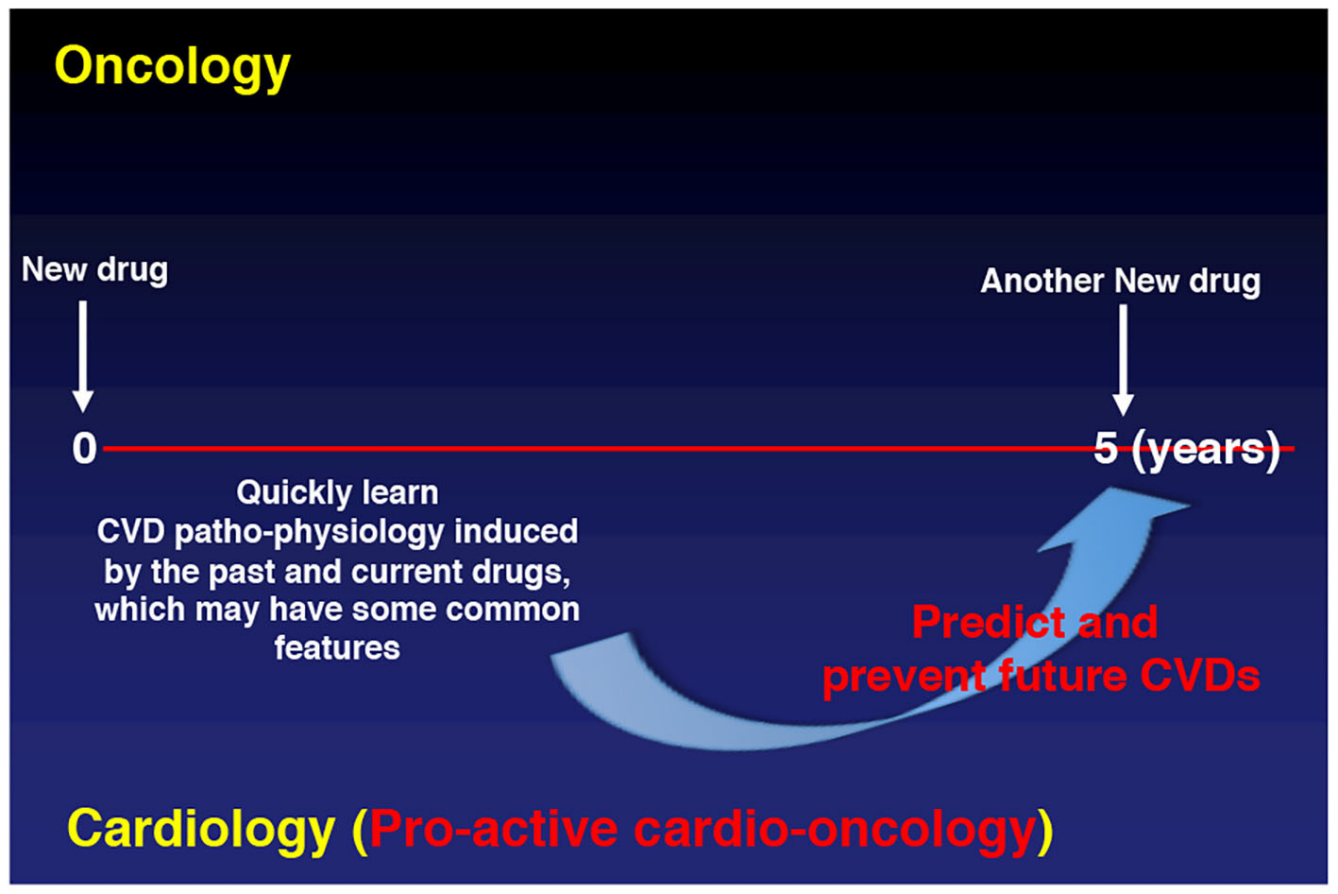
Future.

Providing the evidence base for these practice and education related efforts is of utmost importance. It calls for research that will provide new insight, mechanistically, translationally, and clinically, and help transition cardio-oncology from a “Band-Aid” to an evidence-based practice approach. These research efforts should cover the entire continuum of cancer care, from before, to the during, and the after cancer therapy with most optimal prevention, surveillance, and treatment. Furthermore, at the time of drug development itself, its potential for cardiovascular toxicity should be carefully evaluated such that at the time of its introduction one can be prepared to expect and address emerging issues. Big data and artificial intelligence may aid in this regard and steer into a bright future. As outlined herein, what is needed are more proactive approaches to change the cardio-oncology practice from “dealing with the common final CV adverse effects” by merely following the oncology practice, to an evidence-based practice by learning the pathophysiology and principles of cardiovascular toxicities across cancer treatments (Figure 3).

Changing paradigms, changing times, and changing minds, this is how we see the past, present, and future of cardio-oncology.

## Author Contributions

All authors listed have made a substantial, direct and intellectual contribution to the work, and approved it for publication.

## Conflict of Interest

The authors declare that the research was conducted in the absence of any commercial or financial relationships that could be construed as a potential conflict of interest.
